# Complete Heart Block Secondary to COVID-19

**DOI:** 10.7759/cureus.14970

**Published:** 2021-05-11

**Authors:** Amr Mohamed

**Affiliations:** 1 Internal Medicine, Rochester Regional Health, Rochester, USA

**Keywords:** complete heart block, covid19

## Abstract

In our everyday practice with COVID-19 patients, we noticed that they are not tachycardic even when critically ill and shocked on pressors they are bradycardic. Most of them are in sinus rhythm. However, some of them go into advanced degree heart block and had cardiac arrest related to complete heart block. A 72-year-old female with a history of hypertension and diabetes admitted with COVID-19 bronchopneumonia. The hospital course has been complicated by respiratory failure requiring mechanical ventilation and septic shock requiring norepinephrine. It was noted that she is bradycardic in the 50s in sinus rhythm, her heart rate dipped down to 30 for about six seconds, and her telemetry strip showed evidence of complete heart block, and then she was back in sinus rhythm. The decision was made to follow-up, given that it was for a short time and did not recur. The next day she had cardiac arrest related to a complete heart block, and a temporary trans-venous pacemaker was placed, Later when her condition improved, her heart rate started recovering gradually. At first, she was in junctional tachycardia, and one week later, she was back in sinus rhythm. The temporary pacemaker was removed, and she was discharged on a ZIO monitor for two weeks with no further evidence of bradycardia. Bradycardia in COVID-19 patients is frequently reported. It is not always benign and should be managed promptly when there is evidence of advanced heart block.

## Introduction

In our ICU at Rochester General Hospital, we noticed that most COVID patients are bradycardic. Bradycardia sometimes even affects our decision when choosing what vasopressor to use while they are in shock. Bradycardia is frequently described in the literature, but complete heart block and cardiac arrest related to COVID are not frequently delineated. We are shining the light on a case of complete heart block related to COVID-19.

## Case presentation

**Figure 1:**
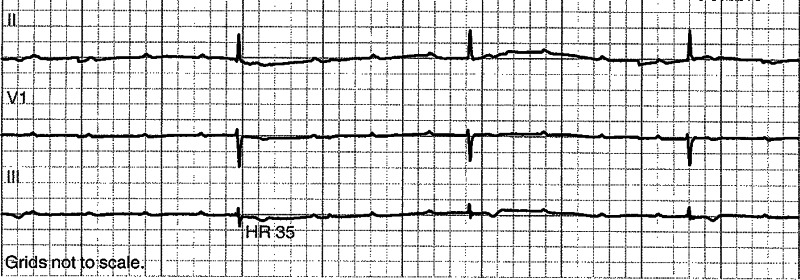
Telemetry strip showing AV dissociation with complete heart block.

A 72-year-old female with a past medical history of hypertension and diabetes was admitted with COVID-19 bronchopneumonia. Her hospital course has been complicated by respiratory failure requiring mechanical ventilation, septic shock requiring norepinephrine. During the admission, she was bradycardic in the 50s in sinus rhythm. Her heart rate dropped down to 30 for a short time of about six seconds. The rhythm strip showed AV dissociation consistent with complete heart block, as shown in Figure 1.

The bradycardia episode did not last long enough to obtain 12 lead EKG. She was then back in sinus rhythm. Her transthoracic echocardiography was unremarkable with normal ejection fraction and no segmental wall motion abnormality, brain natriuretic peptide (BNP) within normal limits, troponin I elevated up to 50 ng/ml. Troponin elevation was attributed to demand-mediated myocardial ischemia.

**Figure 2:**
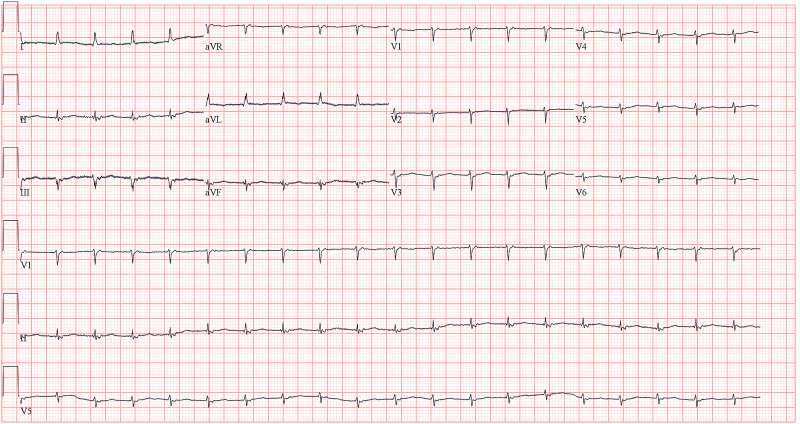
12 lead surface EKG showing junctional tachycardia.

The decision was made to follow up, given that the complete heart block was for a short time and did not recur. The patient was not on any medication or sedation that can cause bradycardia and was oxygenating well on the ventilator. The next day she had pulseless electrical activity (PEA) cardiac arrest related to complete heart block, and return of spontaneous circulation (ROSC) was achieved after three minutes, and a temporary trans-venous pacemaker was placed, and she was paced most of the time because of persistent bradycardia and complete heart block. Later, when her condition improved and she was extubated, her heart rate started recovering gradually. At first, she was in junctional tachycardia, as shown in Figure 2, and there were no further bradycardia episodes.

**Figure 3:**
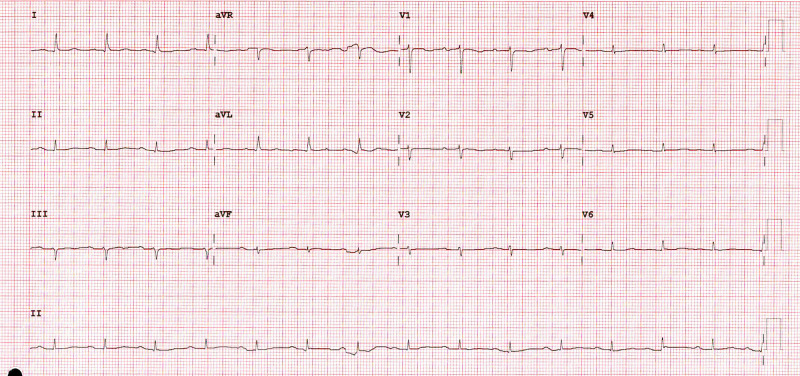
12 lead surface EKG showing sinus rhythm with borderline first-degree heart block.

She remained in hospital because of poor physical condition and need for rehabilitation placement, and all that time, she was on continuous telemetry. One week later, she was back in sinus rhythm, as shown in Figure 3. the temporary pacemaker was removed, and she was discharged on a ZIO monitor for two weeks with no further evidence of bradycardia.

## Discussion

Multiple bradyarrhythmias and tachyarrhythmias had been described to happen in COVID-19 patients. Sinus bradycardia also had been described frequently [1], advanced heart block is now seen in the ICU related to COVID-19 and needs to be put on the radar because it is a fatal bradyarrhythmia.

The pathogenesis of bradycardia remains poorly understood; multiple proposed mechanisms include increased vagal tone [2], direct COVID involvement to the heart's conduction system, or even COVID myocarditis. We wonder if it is the exact pathophysiology as giant cell myocarditis, as it is also associated with complete heart block [3].

The treatment is still according to advanced cardiac life support (ACLS) guidelines for symptomatic bradycardia. According to the European Society of Cardiology/American Heart Association (ESC/AHA) guidelines, if an advanced degree of heart block is found, for example, Mobitz type II and higher or if the bradycardia is causing hemodynamic instability or is symptomatic, this became very unstable. Temporary pacing should be done to prevent potential deterioration resulting in hemodynamic instability or cardiac arrest, as seen in this case. In our opinion, the complete heart block could be related to COVID myocarditis given that the patient had bradyarrhythmia with significant troponin elevation in the absence of segmental wall motion abnormality and that it resolved with the resolution of COVID as demonstrated in this case but still difficult to prove and needs to be further studied.

## Conclusions

The main message is to keep in mind that despite that bradycardia in COVID-19 patients is frequently reported, it is not always benign and should be managed promptly when there is evidence of advanced heart block.
